# 

**Published:** 1849-01

**Authors:** 


### NOTICE TO MANUFACTURERS.

At the last annual meeting of the Mississippi Valley Association of Dental Surgeons, the following resolution was passed, and the Corresponding Secretary directed to communicate it to the different manufacturers throughout the United States:

*Resolved,* That this society offer to the manufacturer a gold medal worth twenty dollars, for one hundred of the best teeth offered, including specimens of gum teeth, of molars, bicuspidati, and common plate and pivot teeth in equal proportions; and that for the second best, of the same amount and character, be presented a silver medal worth ten dollars.

The teeth may be sent to care of Corresponding Secretary, who will lay them before the Committee, and the manufacturer's name will not be made known until after suitable tests have been made and the premiums awarded. The lots not receiving premium, will be subject to the order of the senders; and no names will be announced but of those who receive premiums. We hope for a lively competition.

The teeth must be received before the first of Sept. 1849.

E. TAYLOR, Cor. Sec.

### AMERICAN JOURNAL OF DENTAL SCIENCE.

THE October No. of this valuable work is just received. By a hasty glance at its contents, we should judge it to be of more than ordinary interest. The lengthy report on practical dentistry, by C. O. Cone, D. D. S., of Baltimore, presented at the last meeting of the American Society of Dental Surgeons, we judge from the arrangement, to be an excellent compend of valuable matter.

We also notice that "Hill's Stopping," is working itself into the Journal, if not into the Teeth.

An article on Tic Douloureux, by Dr. Hullihen, of Wheeling, we shall at our first leisure, peruse with pleasure.

We also notice an article on Decay in Teeth, by J. Lee, M. D., of Camden, S. C.

Also notice of the ninth annual meeting of society of Dental Surgeons. The proceedings New York do., and Pennsylvania do.

We hope in our next to refer to these more at length. We received the work too late to give even a brief abstract of these meetings, or more than take a glance at the contents.—CIN. ED.

---

### AGENTS FOR THE REGISTER.

THE last meeting of the Mississippi Valley Association of Dental Surgeons, authorized the Editors of the Register to appoint such agents as they may see proper, for the procuring of subscribers, and to pay them the usual per cent. for their services.

We shall, therefore, send a few copies of No. 1 and 2, Vol. 2, of the Register, to the following individuals, who are authorized to act as agents for the work.

Copies of volume 1st will be supplied to either of these gentlemen, or to any subscriber, whenever ordered.

AGENTS.

Dr. ASAHEL JONES, Dental Depot 263 Broadway, New York.
" S. W. STOCKTON, Dental Depot 116 Chesnut st., Philadelphia.
" J. M. BROWN, Dental Depot, Melodeon building, 4th st., Cin.
Mr. JOSEPH BURNETT, Druggist, No. 33 Tremont Row, Boston.

